# Prediction of violent reoffending in people released from prison in England: External validation study of a risk assessment tool (OxRec)

**DOI:** 10.1016/j.jcrimjus.2023.102061

**Published:** 2023-05

**Authors:** Gabrielle Beaudry, Rongqin Yu, Owen Miller, Lewis Prescott-Mayling, Thomas R. Fanshawe, Seena Fazel

**Affiliations:** a Department of Psychiatry, University of Oxford, Oxford, UK; b Thames Valley Police, Kidlington, UK; c Nuffield Department of Primary Care Health Sciences, University of Oxford, Oxford, UK; d Oxford Health NHS Foundation Trust, Oxford, UK

**Keywords:** Risk assessment, Clinical prediction model, Validation, Prison, Recidivism, OxRec

## Abstract

We aimed to externally validate the Oxford Risk of Recidivism (OxRec) tool to estimate 1- and 2-year risk of violent reoffending in people released from prison in England.

We identified individuals using administrative data shared between official prison and police services. We extracted information on criminal history, clinical and sociodemographic risk predictors, and outcomes. Predictive ability was examined using measures of calibration and discrimination for predetermined risk thresholds.

In total, 1770 individuals (median age = 33 [IQR 27–40]; 92% were male) were identified. 31% and 43% reoffended within 1 and 2 years, respectively. Discrimination was good, with AUCs of 0.71 (95% CI: 0.69-0.74) for 1 year and 0.71 (0.68-0.74) for 2-year follow up. At a pre-specified threshold of 40% for 2-year risk, sensitivity was 77% (74%–80%), specificity 54% (51%–58%), PPV 56% (53%–59%) and NPV 76% (73%–79%). Simple model validation found a systematic underestimation of the probability of reoffending. However, after updating the model, calibration was good.

External validations of risk assessment tools can be conducted using linked data between prison and police, and may require recalibration before implementation. In this validation, OxRec had good performance on discrimination and calibration measures. It can be considered to be used to improve decision-making about risk of serious offending and the allocation of resources.

## 1 Introduction

More than 11 million people are currently estimated to be in prison worldwide (Fair & Walmsley, 2021), with 2 year rates of reoffending typically around 40–60% in high income countries (Yukhnenko, Sridhar, & Fazel, 2019). Preventing reoffending is thus a priority for the criminal justice system as part of public safety initiatives (The Lancet Public Health., 2022). Thus, using prediction models (also termed risk assessment tools) have gained importance in criminal justice to identify high risk persons and inform sentencing, supervision, and rehabilitation, particularly for approaches that are linked to interventions targeting modifiable factors included in these tools (van Ginneken, 2019). Another rationale for their widespread use is that these tools may provide a more accurate and reliable risk assessment than unstructured clinical judgement (Ægisdóttir et al., 2006), and provide consistency within and across services. Applications of these tools have been advocated as an evidence-based approach towards treatment allocation, particularly for people in prison and on release from prison with psychiatric disorders and substance misuse where there are treatments that could modify risk (Fazel, 2019; Yu et al., 2022).

However, despite the high number of structured risk assessment tools currently in use (Singh et al., 2014), few have been validated in settings that differ from those in which initially developed. Existing ones, for example, LSI-R, HCR-20 and COMPAS tend to be underpowered, have a high risk of bias, and do not report key performance measures, particularly calibration (or the agreement between expected and observed risk). Few high quality external validations exist (Fazel et al., 2022). Thus, there is an imbalance between model development and external validation (which is also the case for prognostic tools in medicine) (Ramspek, Jager, Dekker, Zoccali, & van Diepen, 2020).

In the few external validations of criminal risk assessment tools, a common problem is that studies only report the AUC as a performance measure. However, this is difficult to interpret as a standalone metric since it measures how well the model discriminates across all possible cut-offs, most of which are not clinically relevant (Alba et al., 2017; Mallett, Halligan, Thompson, Collins, & Altman, 2012). International research guidelines have recommended reporting a fuller set of performance measures, including false positive and negative rates, and calibration measures to inform evidence-based clinical decision-making (Collins, Reitsma, Altman, & Moons, 2015; Gulati et al., 2022; Steyerberg, 2009). For example, one risk tool—the Offender Assessment System (OASys) and the related OASys Violence Predictor (OVP)—is routinely used by probation officers in England and Wales to assess individual needs and future risks (Howard, 2006). To date, the published performance of this tool has been limited to discrimination measures (Debidin, 2009; Howard & Dixon, 2011, 2012). Furthermore, most risk tools are not implemented in practice and lack external validation in settings different from where they were originally developed (Fazel, Burghart, et al., 2022).

To address these limitations, a new and scalable violence risk assessment tool, Oxford Risk of Recidivism (or OxRec), was developed (Fazel et al., 2016). OxRec was derived and validated with data from population-based registers from Sweden to estimate the risk of violent reoffending following release from prison. This tool was developed with high quality methods, including a prespecified protocol, multivariable regression models, internal and external validations, and reporting of key performance measures, including discrimination and calibration. More specifically, OxRec was developed using risk factors in three main domains associated with recidivism: sociodemographic, criminal history, and clinical. Within these domains, risk factors were identified based on review literature (Bonta, Law, & Hanson, 1998; Witt, Van Dorn, & Fazel, 2013), and then tested in multivariable Cox regression models to identify predictors that retained statistical significance for violent recidivism. The final prediction model had 14 items in sociodemographic (sex, age, immigrant status, civil status, education, employment, disposable income, neighbourhood deprivation), criminal history (length of incarceration, violent index offence, previous violent crime), and clinical domains (alcohol or drug use disorder, any mental disorder, any severe mental disorder) (Fazel et al., 2016). This included primary research that was more recent that showed that clinically diagnosed mental and substance use disorders are associated with recidivism outcomes (Chang, Larsson, Lichtenstein, & Fazel, 2015).

Risk factors were then assigned to one of three groups based on the strength of the evidence supporting their association with the outcome (i.e. violent reoffending) (Fazel et al., 2016) and then weighted according to their effect sizes in the Cox regression model in the final model. OxRec provides risk probabilities and also a categorical risk level (low/medium/high) based on prespecified probability scores to help decision-making in criminal justice and mental health, and assist with identifying individuals who would benefit from linkage with community healthcare and addictions services. OxRec is intended to complement professional judgement, and freely accessible online (https://oxrisk.com/oxrec-9/).

In this study, we report an external validation of OxRec to estimate violent reoffending risk in people released from prison in England. Our objective was to investigate whether the OxRec tool—initially developed to predict risk of offending in individuals released from prison in Sweden—is applicable to a cohort of previously incarcerated individuals in England. This is a necessary step for its potential use in a UK setting. We report the first such UK study and using routinely-collected police data to capture reoffending information. The use of risk assessment tools in police settings is rare as capacity is limited. Hence, a scalable brief tool could benefit police services, and provide more linkages with other stages of the criminal justice system.

## 2 Methods

### 2.1 Study design and participants

We identified the sample using administrative datasets shared between His Majesty’s Prison and Probation Services (HMPPS) and police services. This dataset contains all movements in and out of the HMPPS establishments. We selected individuals who were present in both HMPPS and Thames Valley Police’s crime recording system (using the NICHE Record Management System). More specifically, we used data on criminal records that pooled information from people released from prison into the Thames Valley Police Force area (i.e., the three counties of Buckinghamshire, Berkshire, and Oxfordshire) in England aged 18 years and older (or treated as adults by the law). The study sample included persons released from prison between April 1, 2017, and March 31, 2018, in a region of around 2.4 million population that includes both metropolitan and non-metropolitan areas. The sample size was based on prognostic modelling guidelines that recommend at least 100 outcome events (i.e. violent reoffences for this study) for validation studies to be adequately powered (Vergouwe, Steyerberg, Eijkemans, & Habbema, 2005).

Individuals entering the national Criminal Justice Information Service are informed about secondary usage of data, thus the standard requirement of written informed consent was waived. We used existing routinely collected police data. Ethical approval was granted by the Central University Research Ethics Committee (CUREC) of the University of Oxford (R44562/RE001). This study complies with the Transparent Reporting of a multivariable prediction model for Individual Prognosis or Diagnosis statement (TRIPOD) (Collins et al., 2015).

### 2.2 Outcomes

We obtained information on reoffending outcomes from the NICHE Record Management System (a police register used to record offences that are reported and investigated by the police force). The following outcome-related data were recorded: (i) suspected of a violent offence by police, (ii) convicted of a violent offence, (iii) did not reoffend, and (iv) been lost to follow-up. At 1 and 2 years following prison release, outcomes were reported in binary terms (i.e. ‘violent reoffence’ vs. ‘no violent reoffence’). The definition of violence was a standard one used by police that included two categories of ‘assault violence or threat of violence’ (i.e. any interpersonal violence and violent threats) and ‘rape and penetrative sexual offences’ (i.e. any contact sexual crimes). People released from prison were followed up from the date of their release until the outcome first occurred or the end of the study (within 24 months).

### 2.3 Predictor variables

We examined the predictor variables based on the original version of OxRec. Several modifications to variable definitions were required to adapt predictors to the local context (Supplementary Table 1). For instance, sex (assigned at birth) was replaced by gender since only data on the latter was accessible. Immigrant status was based on nationality (instead of first or second-generation immigrant status in the development sample). Neighbourhood deprivation was calculated using the Index of Multiple Deprivation (IMD; 2019), which is the official measure of deprivation for small areas in England. More specifically, the last valid residential or family home address before the release date was linked with the Lower-layer Super Output Areas (LSOA) to determine the relevant IMD decile (Noble et al., 2019). Data on clinical variables (i. e. alcohol and drug use disorder, any mental disorder) were based on warnings recorded by police officers and custody staff in the NICHE Record Management System, rather than formal diagnoses according to ICD or DSM classifications. We could not include the following two predictors for which data were not available: highest education and disposable income. In addition, a second-level predictor, any severe mental disorder, was not available, which is only scored if someone meets ‘any mental disorder’ criteria. These differences had potential implications for calibration and were therefore considered in the validation process.

### 2.4 Statistical analyses

Baseline characteristics were detailed by counts and percentages for categorical variables, and as median (IQR) for continuous variables. These were compared to those of the Swedish derivation sample. OxRec was validated using an incremental strategy (Steyerberg, 2009; Su, Jaki, Hickey, Buchan, & Sperrin, 2018), which were used in previous studies (Beaudry, Yu, Alaei, Alaei, & Fazel, 2022; Fazel et al., 2019). This approach involves three potential steps (1) simple validation, by applying the original prediction model (all model coefficients at their original value, including the baseline risk estimate); (2) updating the baseline risk and calculating a multiplicative recalibration value; (3) performing a selective re-estimation of coefficients for individual predictors. Each phase evaluates prognostic performance, and progression to the next step is only necessary in the event of poor performance (Steyerberg, 2009). The full validation protocol (including recalibration with a Cox proportional hazards model) has been published online as a part of the OxRec validation in the Netherlands (Fazel et al., 2019).

We examined several model performance indicators to determine the predictive ability of the model in terms of discrimination (the model’s ability to separate out individuals who have reoffended from those who have not), and calibration (the level of agreement between observed and expected outcomes). These indicators included the area under the receiver operating characteristic curve (AUC–ROC), or *c* index, as well as sensitivity, specificity, and positive and negative predictive values (PPV and NPV) (for discrimination); and the Brier score, calibration slope and calibration-in-the-large (CITL) (for calibration), defined as the ratio of prevalence of observed to predicted events (Fazel, 2019; Steyerberg, 2009). We selected cut-off scores that were easy to interpret and close to the baseline rates to calculate sensitivity, specificity, NPV, and PPV values.

We used multiple imputation by chained equations to replace missing values for immigrant status, length of incarceration, violent index offence, civil status, and employment. Twenty imputations were carried out based on recommended practice (Steyerberg, 2009; White, Royston, & Wood, 2011). Multiple imputation takes into account all available data (both from complete and incomplete cases) to construct several imputed data in which the missing values are replaced (Steyerberg, 2009). Moreover, we averaged out the predictors that were missing completely (i.e. highest education, disposable income, and any severe mental disorder), by assigning all subjects their average value from the derivation sample. All analyses were conducted using STATA software (version 17.0, StataCorp, College Station, TX, USA).

## 3 Results

### 3.1 Baseline characteristics

The validation cohort included 1770 people released from prison in England. The median (IQR) age was 33 (27–40) years, and males accounted for 92% of the sample. The proportion of missing data on predictors varied from <5% (immigrant status, length of incarceration, violent index offence) to 15% (employment status). We compared baseline characteristics between the validation (England) and the original derivation (Sweden) cohorts. The prevalence for most of the variables was similar, but there were differences. The length of incarceration appears on average longer in the validation cohort (31% over 24 months in the validation sample vs. 4% in the original derivation sample). A higher percentage of individuals were in employment at the time of incarceration (63% vs. 25%) (Table 1). Follow-up data were obtained from all participants (Supplementary Table 2). Base rates of violent reoffending (i.e. including both suspected and convicted for violent crime) for the two time points (1 and 2 years) were higher in the validation cohort than in the original derivation study. The primary outcome, violent reoffending, occurred in 31% (550 of 1770; 1-year follow-up) and 43% (765 of 1770; 2-year follow-up). This compares with violent reoffending rates of 12% at 1 year and 21% at 2 years in the original derivation sample (the latter which solely included convictions rather than suspicions and convictions in the current study).

### 3.2 Model performance and recalibration

When refitting the OxRec model in the validation data, there was some miscalibration for both time points (CITL = 1.72; [1 year]; CITL = 1.73; [2 years]). The observed violent reoffending probabilities were systematically higher (by around 20%) than expected, where the actual risk was 2–3 times what the model predicted. Thus, we updated the baseline survival function and recalibrated the linear predictor to align the predicted and observed survival probabilities for all risk deciles (Table 2). This step improved OxRec’s calibration and the revised model showed good calibration (i.e. calibration in the large was null for both time points, Supplementary Table 3). Despite this, calibration plots indicated a slight overestimation of violent reoffending risk in lower risk deciles and an underestimation in the higher deciles. The effects of predictors were similar in the development and validation samples, thus no re-estimation of the original coefficients was required (Supplementary Table 4).

**Fig. 1:**
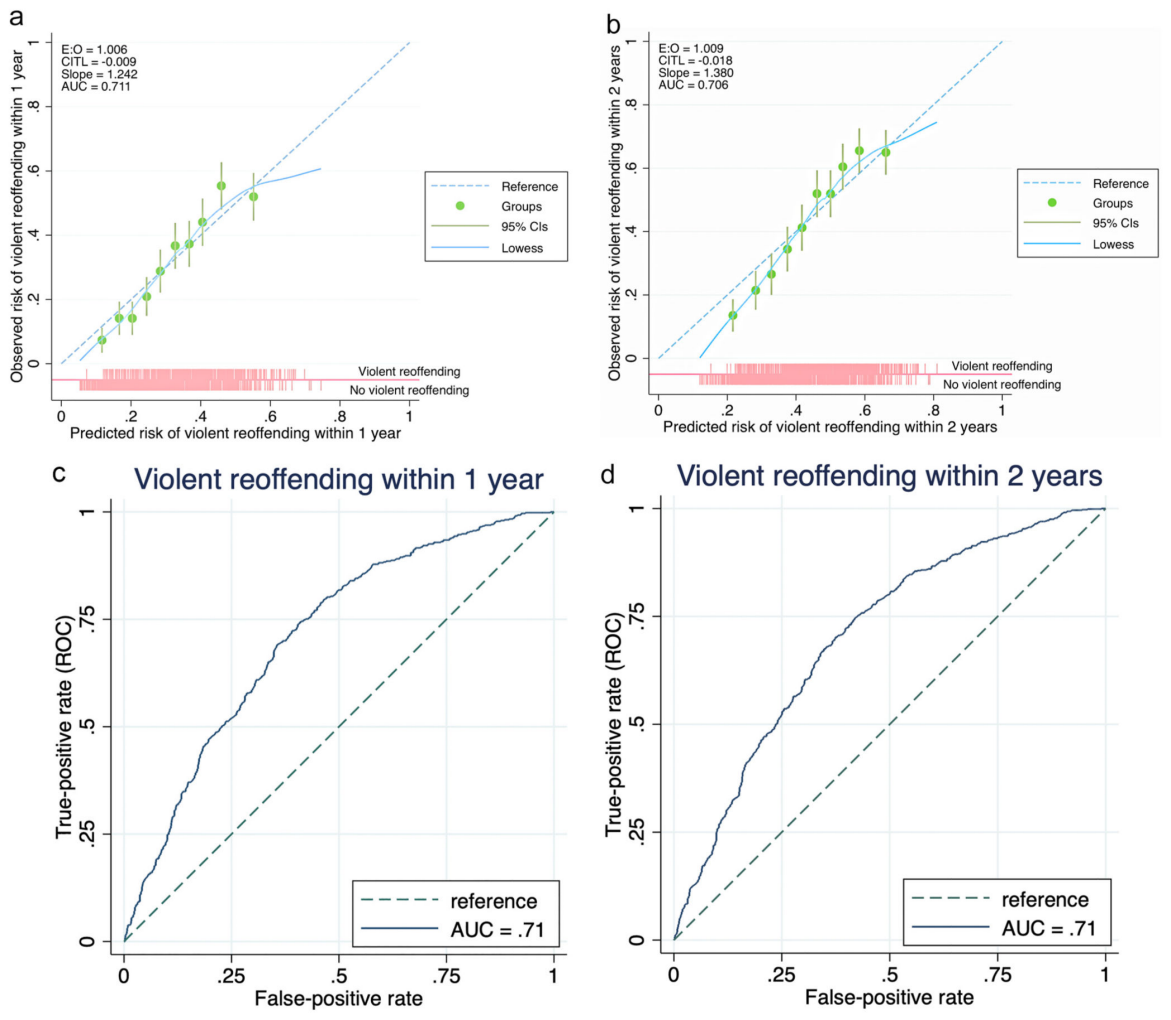
Calibration plots of the OxRec model in a cohort of 1770 people released from prison: a: 1-year violent reoffending. b: 2-year violent reoffending. Receiving-operating characteristic curve (ROC) for external validation of OxRec model: c: 1-year violent reoffending. d: 2-year violent reoffending. AUC = area under the curve; CITL = calibration in the large; E:O = ratio of expected to observed outcomes;

ROC curves and calibration plots for the updated OxRec model are shown in Fig. 1. As for discrimination, AUCs for violent reoffending at 1 and 2 years were 0.71 (95% CI 0.69–0.74 [1 year]; 0.71 (0.68–0.73) [2 years]). For risk of violent reoffending at 1 year (using a 30% risk cut-off), sensitivity was 74% (95% CI 0.70–0.78) and specificity was 59% (95% CI 0.56–0.62), whilst positive and negative predictive values were 45% (95% CI 0.42–0.48) and 83% (95% CI 0.81–0.86), respectively. At 2 years, using a 40% cut-off, sensitivity was 77% (95% CI 0.74–0.80) and specificity was 54% (95% CI 0.51–0.58). Positive and negative predictive values were 56% (95% CI 0.53–0.59) and 76% (95% CI 0.73–0.79), respectively. Discrimination is presented for additional risk cut-offs, including for 10%, in Table 3.

## 4 Discussion

We have externally validated a risk assessment tool for reoffending (OxRec) in a cohort of 1770 people released from prison. One and two-year violent reoffending rates were 31% and 43%, respectively, which was consistent with national recidivism rates (Yukhnenko et al., 2019). The final model yielded good discrimination (with an AUC of 0.71). Furthermore, unlike other risk assessment tools, we have updated the OxRec model by recalibration following a pre-determined protocol. The updated model demonstrated good calibration. In this study, we have also demonstrated how police data can be used to estimate reoffending outcomes, which allows for more comprehensive data for recidivism.

Two main implications follow. OxRec identified individuals at elevated risk of violent reoffending using cut-offs of 30% for 1-year risk and 40% for 2-year risk accurately (i.e. identifying 74% and 77% of people who violently reoffended within 1 and 2 years, respectively). These individuals could be prioritised for additional support on release given limited resources in criminal justice and likely benefits to the individual, such as psychological interventions directed at modifiable risk factors (e.g. substance misuse) and service-related ones, such as ensuring linkage with community mental health services.

Second, the tool also identified individuals at low risk of violent reoffending based on the same cut-offs (i.e. 59% and 54% who did not violently reoffend at one- and two-year follow-up periods). This could assist with decarceration efforts, whereby individuals in this low-risk group could be released with adequate supervision and treatment in the community (Fazel, Burghart, et al., 2022). There will be a balance between sensitivity and specificity in determining what cut-offs to use in practice, or to avoid categories and use probability scores alone. From a population and policy perspective, higher sensitivity (e.g. 77% for the primary outcome at 2 years), as found here, will be more important than specificity—in other words, missing those who will reoffend (false negatives or 1-sensitivity) is likely to be less acceptable than false positives (1-specificity). False positives, reported above 40% in the current study, can be tolerated if they lead to non-harmful consequences, such as adding treatments and interventions that target individual needs (Pickard & Fazel, 2013).

Used in conjunction with professional judgement, evidence-based risk assessment tools such as OxRec have the potential to reduce economic costs to the criminal justice system and beyond, allowing for criminal justice agencies to assist released prisoners to reintegrate safely into society. The risk score obtained could be used to supplement professional judgement. In addition, as OxRec could be run on existing administrative data without the need for additional information, which would lend itself to provide automated risk assessments that are available alongside the release information that the police usually receive. This is particularly the case for individuals who are released from prison, some of whom will move to a new region, where it is not feasible to conduct clinically or interview-based risk assessments. In the UK, high risk people are managed by the Multi-Agency Public Protection Arrangement (MAPPA), a national statutory process for managing people convicted of serious violent and sexual offences, which meets monthly and is jointly chaired by police and probation. In this context, OxRec could be used to identify people for appropriate management under MAPPA and, as it could be implemented at release from prison, depending on sentence or risk, identify those who should be actively managed under more intensive supervision (i.e. MAPPA categories 2 and 3). Such arrangements under MAPPA are consistent with national public health and safety initiatives (Nacro National Assocation for the Care and Resettlement of Offenders, 2022), which require police, other criminal justice agencies and community health to work together to reduce such crimes.

Whilst existing models may have lower accuracy in external settings, validating a model developed using high quality methods (such as OxRec) saves time and resources compared to developing a new tool for each setting. OxRec had a similar or higher AUC than other risk prediction instruments, most of which take considerably longer, that are used in criminal justice (Fazel et al., 2022). We also found similar associations of risk factors in OxRec with violent reoffending with original study (Fazel et al., 2016). The base rate of violent reoffending in the English sample was more than double that of the Swedish sample (i.e. 31% vs. 12% [for 1 year] and 43% vs. 21% [for 2 years]), and therefore predicted OxRec probabilities of violent reoffending were underestimated compared to observed reoffending rates, before model updating. This difference reflects using a more sensitive outcome definition (i.e. police suspicions and official crime convictions in this validation vs. solely crime convictions in the Swedish sample), as official recidivism statistics are similar in both countries (Yukhnenko et al., 2019). The initial calibration would likely have been better if a similar outcome was used, underscoring the importance of outcome definition in validation studies. Moreover, differences in baseline characteristics such as employment and previous violence might also explain why the model needed recalibration.

The findings of this study further suggest that OxRec is transportable across different populations and geographical settings following recalibration, as evidenced by good discrimination and calibration in several external validations including the present study, and others conducted in Tajikistan (Beaudry et al., 2022) and the Netherlands (Fazel et al., 2019). Despite the considerable differences between Tajikistan, a lower-middle income country, and the setting in which the initial model was developed (Sweden), its performance remained robust without significant modifications (such as the re-estimation of risk factor coefficients). As indicated by the AUC, the model achieved similar levels of discrimination in the English sample (0.71) to that of the Tajik (0.70) and Dutch samples (0.68). This suggests that the OxRec model is generalizable to other countries.

In comparison with OVP—currently being used by the National Offender Management Service in England and Wales for risk and needs assessment—the OxRec tool achieved similar levels of predictive performance when considering solely the AUC (0.71 [OxRec] vs. 0.72 [OVP]) (Howard & Dixon, 2012). However, other key measures of OVP’s predictive performance are poorly reported and many are lacking. Measures of OVP discrimination beyond the AUC (i.e. sensitivity, specificity, PPV, NPV) were calculated based on arbitrary classification risk thresholds, solely selected to match the distribution of another tool’s categories (i.e. the V scale of Risk Matrix 2000 [RM2000/V]) for comparison purposes (Thornton et al., 2003). Calibration performance for the OVP has not been reported, despite being necessary to ensure that predictions are not misleading (Van Calster et al., 2019), and recommended by methodological guidelines for prediction modelling (Collins et al., 2015). In contrast, OxRec’s external performance was evaluated using pre-specified risk thresholds (for discrimination) and appropriate measures and visualisations (for calibration). OxRec also has the advantage of including fewer predictors than OVP, and most of OxRec’s factors are common and routinely collected, thereby allowing for its use in practice. Hence, it can be easily calculated (in <10 min) and incorporated into existing risk assessment practices by probation officers. Another key strength of this validation study is that it used data from a routine source of information used by most UK police forces, the NICHE Record Management System. This suggests that the validated tool could be transferable to other UK criminal justice agencies.

Study strengths include combining the accuracy of probabilistic predictions with a relatively simple prediction tool. The contribution of predictor variables to the outcome is clear, and their relationship is interpretable. Another strength of OxRec is transparency, with the full model including the coefficients, being reported (Fazel et al., 2016). This is lacking in most tools in criminal justice where the original development studies are typically not published and where actuarial tools do not publish their full models (Fazel, Burghart, et al., 2022). We have provided multiple performance indicators for independent examination, critical appraisal and reproducibility of the model and the methods used to validate it. Transparency in model development and validation is critical given the possible ramifications for justice-involved individuals and public health and safety, and to ensure a fair criminal justice system (Fazel, Sariaslan, & Fanshawe, 2022). Overall, OxRec appears to be a promising tool for predicting violent reoffending in people released from prison, because it has better predictive accuracy compared to other tools, shorter administration time, and is transparent (with the study protocol, the final model, formula and coefficients making up the risk scores published) (Fazel, Sariaslan, & Fanshawe, 2022).

Several limitations should be noted. First, since NICHE includes information on both crimes solved and those under investigation, which was used as the outcome. At the same time, this is a more sensitive measure than convicted re-offending and captures the population-level effects of violent behaviour as police investigations (as distinct from police charges) require a threshold that conviction is more likely than not. Second, some predictors were missing from the validation dataset (i. e. highest education and disposable income), whilst proxies were used for some others (i.e. clinical risk factors). For entirely missing predictors, although they had small effects on the predicted outcomes in the original model, all participants were assigned an average value (equivalent to the prevalence in the derivation study), which is the same as incorporating its effect into the estimate of baseline risk (Fazel et al., 2019; Held et al., 2016). To mitigate the impact of missingness for partially missing predictors, we employed multiple imputation, by which plausible values were derived based on other (observed) predictor values (Harel et al., 2017; Janssen et al., 2010). To reduce the possible effects of missing data, future research should strive for using data from linked population-based registers, although access to such data can be costly and restricted. Few countries currently link health and crime registers on a population level, despite the importance of investigating their intersection—a neglected research area for public health and safety. Finally, this study provides no practical guidance on how OxRec could be utilised as a decision-support tool to help guide interventions to reduce violent reoffending. A clinical impact study, in which the feasibility of implementing OxRec, and its impact on current practices, reoffending outcomes and cost effectiveness will need to be studied (and eventually using a trial design to evaluate impact on outcomes) (Labarère, Bertrand, & Fine, 2014). This will allow for examination of how the risk prediction tool could be integrated effectively into existing operational systems and criminal justice workflows, and whether modifiable risk factors in OxRec could be targeted for treatment (Mudumbai & Rashidi, 2021).

## 5 Conclusion

In this real-world external validation of >1700 people released from prison, a recalibrated version of the OxRec tool demonstrated good predictive performance for risk of violent reoffending within 1 and 2 years after release from prison in England. OxRec could be used to supplement professional judgement and assist with linkage to specific interventions and care pathways following prison release. Individuals in higher risk groups could potentially benefit most from effective interventions, given limited resources in criminal justice and health services. The use of evidence-based risk assessment tools has the potential to provide more accurate and consistent decision-making in criminal justice, and also preserve resources and reduce costs due to their scalability. Their potential contribution in reducing criminal outcomes needs to be examined.

## Supplementary Material

F1 - 5

## Figures and Tables

**Table 1:** Baseline characteristics of people released from prison in the English external validation sample compared with the Swedish OxRec development sample.

Variable	English sample (*n* =1770)		Swedish sample (*n* = 37,100)
	Summary	Missing data	
Gender			
*Male*	1622 (92%)		93%
*Female*	148 (8%)		7%
Age	Median 33		Median 36
	IQR 27 to 40		IQR 27 to 46
Immigrant status Length of incarceration	115 (7%)	1 (<1%)	31%
<6 months	658 (37%)		69%
*6–12 months*	253 (14%)	45 (3%)	16%
*12–24 months*	269 (15%)		10%
*≥ 24 months*	545 (31%)		4%
Violent index offence	597 (34%)	18 (1%)	38%
Previous violent crime	1095 (62%)		53%
Civil status			
*Other*	321 (18%)	150 (9%)	35%
*Unmarried*	1299 (73%)		65%
Highest education			
< *9years*	Not available		48%
*9-11 years*			46%
≥ *12 years*			6%
Employment	1116 (63%)	258 (15%)	25%
Disposable income			
*Negative* (*in debt*)			1%
*Zero*			6%
*Low* (<20^th^ *percentile*)	Not available		53%
*Medium* (20^th^–80th *percentile*)			40%
*High* (>*80th percentile*)			1%
Neighbourhood deprivation	0.67 (0.13 to 1.04)		0.39 (–1.18 to 1.47)
Alcohol use disorder	249 (14%)		22%
Drug use disorder	631 (36%)		23%
Any mental disorder	627 (35%)		22%
Any severe mental disorder	Not available		3%

**Table 2:** Recalibrated model formula for the updated version of OxRec.

Sweden	Model formula	Baseline risk coefficient
Violent reoffending (1 and 2 years)	$1-S_{t}\hat{e}xp.(\sum beta\text{×}RF)$	S_1_ = 0.7992 S_2_ = 0.6775
**England**		
Violent reoffending (1 year)	$1-S_{t}\hat{e}xp.(0.6745\times [-0.1838\times 0.4263+-0.4282\times 0.0569+0.5251\times 0.0523+0.5176\times 0.4903+0.3712\times 0.3738+0.4509\times 0.0109+0.0953\times 0.0347+\sum beta\text{×}RF)$	S_1_ = 0.4643
Violent reoffending (2 year)	$1-S_{t}\hat{e}xp.(0.5372\times [-0.1838\times 0.4263+-0.4282\times 0.0569+0.5251\times 0.0523+0.5176\times 0.4903+0.3712\times 0.3738+0.4509\times 0.0109+0.0953\times 0.0347+\sum beta\text{×}RF])$	S_2_ = 0.3509

Note. ‘beta’ and ‘RF’ refer to the model coefficients and risk factors presented in Fazel et al. (2016). The following multiples are adjustments to allow for some predictors being entirely missing in the validation study: highest education (0.4263 [9–11 years] and 0.0569 [≥ 12 years]); disposable income (0.0523 [zero], 0.4903 [low], 0.3738 [medium] and 0.0109 [high]); any severe mental disorder (0.0347).

**Table 3:** Summary of updated model performance of OxRec external validation.

	Prevalence of reoffending	c-index (95% CI)	Risk threshold	Sensitivity	Specificity	PPV	NPV
Violent reoffending (1 year)	31%	0.71 (0.69–0.74)	10%	100% (99–100)	3% (2–4)	32% (30–34)	98% (93–100)
			20%	92% (89–94)	31% (29–34)	38% (35–40)	89% (86–92)
			30%	74% (70–78)	59% (56–62)	45% (42–48)	83% (81–86)
			40%	44% (40–49)	82% (80–84)	52% (48–57)	77% (74–79)
Violent reoffending (2 years)	43%	0.71 (0.68–0.73)	20%	100% (99–100)	4% (3–6)	44% (42–47)	96% (90–100)
			30%	93% (91–95)	27% (24–30)	49% (47–52)	83% (79–87)
			40%	77% (74–80)	54% (51–58)	56% (53–59)	76% (73–79)
			50%	49% (46–53)	77% (74–79)	62% (58–65)	67% (64–69)

Note. Risk thresholds were selected based on original OxRec model and prevalence of violent reoffending in external validation.

## Data Availability

Data for this study are not available, as the participants did not agree for these to be shared publicly. The analysis code can be provided by the authors upon reasonable request.
