# Supplementary material for: Prediction of violent reoffending in people released from prison in England: External validation study of a risk assessment tool (OxRec)
Source: J Crim Justice. Author manuscript; Available in PMC 2024 Dec 6. (PMC7617015; doi:10.1016/j.jcrimjus.2023.102061)
Supplement: F1 - 5 [file EMS173195-supplement-F1___5.docx]

**Supplementary Table 1.** Variable definitions

| **Variable** | **Sweden** | **England** |
| --- | --- | --- |
| Sex | Assigned at birth | Gender (as entered in PINS) |
| Age | Age at release from prison | Age at release from prison, rounded down to full years |
| Immigrant status | First or second generation immigrants (self or either parent born outside of Sweden) | Nationality (as entered in PINS) |
| Length of incarceration | Duration of incarceration for most recent offence | Same definition |
| Violent index offence | Most recent offence was homicide, assault, robbery, arson, any sexual offence (rape, sexual coercion, child molestation, indecent exposure, or sexual harassment), illegal threats, or intimidation | Same definition |
| Previous violent crime | Any conviction for a violent offence previous to most recent offence (i.e. before index offence) | Same definition |
| Civil status | Unmarried vs other (At imprisonment. Other includes married, cohabiting, divorced, and widowed) | Same definition |
| Highest education | Lower secondary, upper secondary, post-secondary | Not included |
| Employment | Employed at incarceration. (Worked for at least 4 hours [based on their income information] during November before incarceration) | Last non-blank occupation before release (taken from Niche Custody) |
| Disposable income | Negative/Zero/Low/Medium/High (Low: <20^th^ percentile, Medium: 20-80^th^ percentile, High: >80^th^ percentile). ‘Low’ and ‘Medium’ disposable income had accounted for 93%. | Not included |
| Neighbourhood deprivation | Principal components analysis of: mean disposable income, % welfare recipients, % unemployed, % divorced individuals, % with only primary school qualifications, % of immigrants (defined as individuals who were not born in Sweden), residential mobility rate, and crime rate. | Last valid residential or family home address before release date, passed through postcode-LSOA calculator and 2019 IMD index quintile. |
| Alcohol use | Diagnosis of alcohol use disorder (lifetime: before or during incarceration – ICD-8: 291, 303; ICD-9: 291, 303, 305A; ICD-10: F10). | Remarks contain “alcoh” (taken from Niche warnings) |
| Drug use | Diagnosis of drug use disorder (lifetime: before or during incarceration – ICD-8: 304; ICD-9: 292, 304, 305 excl. 305A; ICD-10: F11-F19). | Warning type is drugs (taken from Niche warnings) |
| Any mental disorder | Diagnosis of any mental disorder excluding substance use disorders (lifetime: before or during incarceration). | Warning type is mental disorder (taken from Niche warnings) |
| Any severe mental disorder | ICD diagnosis of schizophrenia-spectrum or bipolar disorder (lifetime: before or during incarceration). | Not included |

Note. Data on predictor variables in England were extracted from the PINS and Niche database.

**Supplementary Table 2.** Summary of outcomes in the England and other OxRec samples

| **Outcome** | **England (n = 1,770)** | **Netherlands (n = 9,072)** | **Tajikistan (n = 970)** | **Sweden (n = 37,100)** |
| --- | --- | --- | --- | --- |
| 1 year violent reoffending | 31% | 8% | 15% | 12% |
| 2 year violent reoffending | 43% | 16% | N/A | 21% |

**Supplementary Table 3.** Calibration performance measures for the OxRec tool

|  | **Observed number of events** | **Expected number of events (uncalibrated)** | **Ratio, Expected: Observed**  **(uncalibrated)** | **Ratio of crude event rates, Sweden: England** | **Ratio, Expected: Observed (recalibrated)** | **Brier score** |
| --- | --- | --- | --- | --- | --- | --- |
| 1 year violent reoffending | 550 | 152 | 0.28 | 0.39 | 1.01 | 0.19 |
| 2 year violent reoffending | 765 | 251 | 0.33 | 0.49 | 1.01 | 0.22 |

**Supplementary Table 4.** Risk factors included in the final recalibrated model and their hazard ratios

| **Variable** | **Multivariable adjusted hazard ratio (95% CI)** | |
| --- | --- | --- |
|  | **England** | **Sweden** |
| Sex (female) | 0.75 (0.53-1.07) | 0.51 (0.45-0.57) |
| Age | 0.99 (0.98-1.00) | 0.84 (0.83-0.85) |
| Immigrant status | 0.74 (0.48-1.14) | 0.97 (0.92-1.02) |
| Length of incarceration |  |  |
| <6 months | 1 [Reference] | 1 [Reference] |
| 6-12 months | 0.87 (0.68-1.11) | 0.85 (0.81-0.90) |
| 12-24 months | 0.78 (0.61-1.01) | 0.69 (0.63-0.75) |
| >=24 months | 0.62 (0.49-0.78) | 0.55 (0.48-0.64) |
| Violent index offence | 1.08 (0.90-1.30) | 1.53 (1.46-1.59) |
| Previous violent crime | 2.37 (1.88-2.99) | 2.41 (2.29-2.54) |
| Civil status (unmarried) | 0.87 (0.69-1.11) | 1.08 (1.02-1.15) |
| Highest education |  |  |
| <9 years |  | 1 [Reference] |
| 9-11 years |  | 0.83 (0.79-0.87) |
| ≥12 years |  | 0.65 (0.57-0.75) |
| Employment | 0.94 (0.77-1.15) | 0.68 (0.63-0.72) |
| Disposable income |  |  |
| Negative (in debt) |  | 1 [Reference] |
| Zero |  | 1.69 (1.11-2.57) |
| Low (<20^th^ percentile) |  | 1.45 (0.96-2.19) |
| Medium (20^th^-80^th^ percentile) |  | 1.02 (0.84-1.24) |
| High (>80^th^ percentile) |  | 1.57 (0.92-2.67) |
| Neighbourhood deprivation | 1.10 (0.97-1.24) | 1.03 (1.01-1.04) |
| Alcohol use disorder | 1.41 (1.13-1.75) | 1.41 (1.33-1.49) |
| Drug use disorder | 1.30 (1.09-1.55) | 1.51 (1.44-1.59) |
| Any mental disorder | 1.08 (0.90-1.29) | 1.09 (1.03-1.15) |
| Any severe mental disorder |  | 1.10 (0.99-1.22) |

**Supplementary Figure 1.** Calibration plot and ROC curve before model revision (for violent reoffending within 1 year)




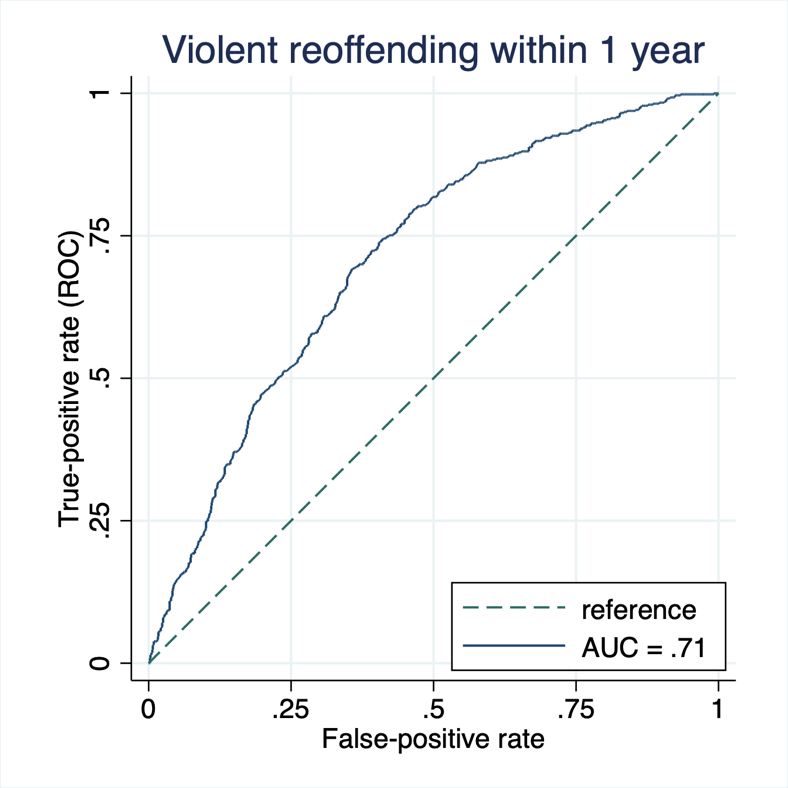


Note. AUC, area under the curve; CITL, calibration in the large; E:O, expected: observed.

**Supplementary Figure 2.** Calibration plot and ROC curve before model revision (for violent reoffending within 2 years)


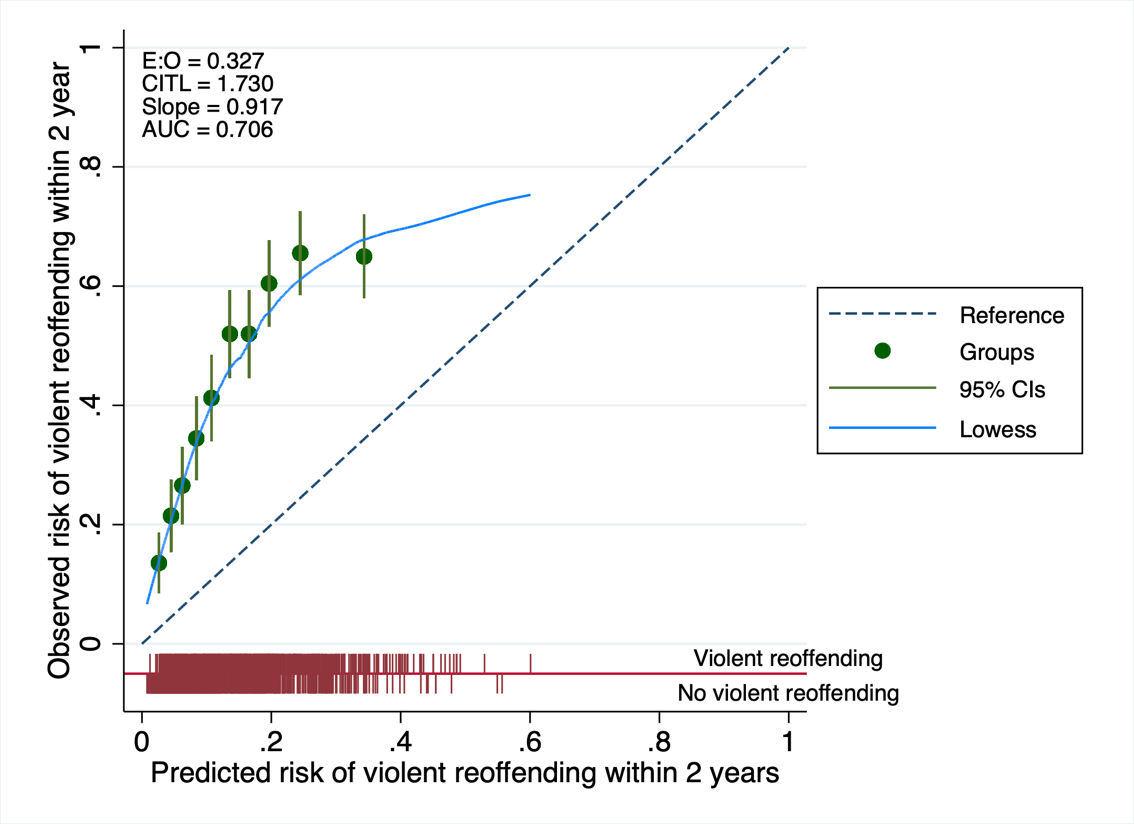

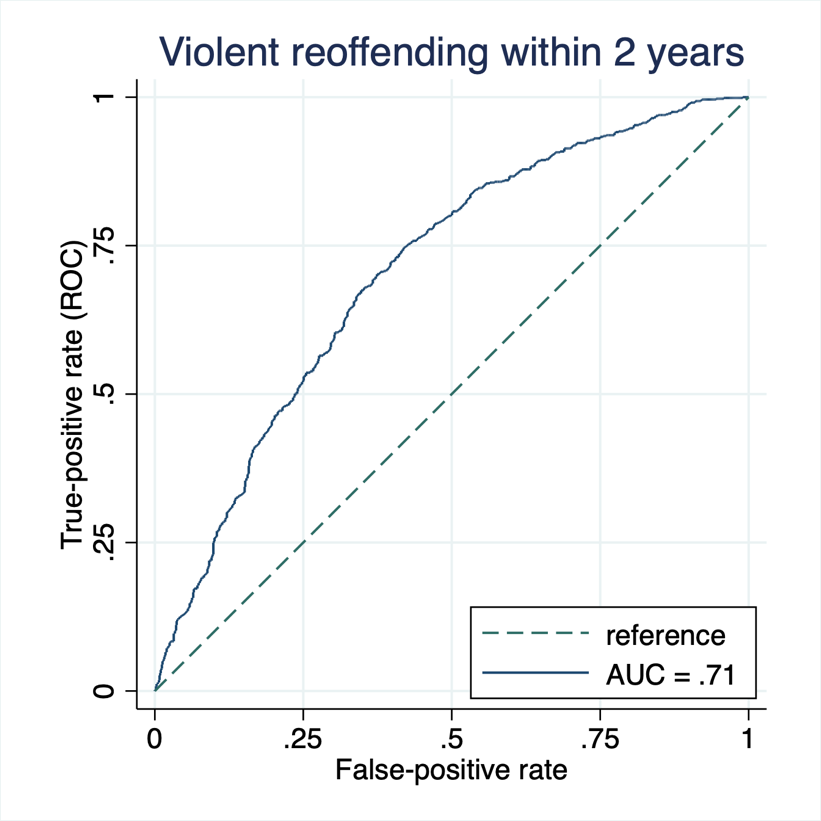


Note. AUC, area under the curve; CITL, calibration in the large; E:O, expected: observed.
